# IFI44L as a Forward Regulator Enhancing Host Antituberculosis Responses

**DOI:** 10.1155/2021/5599408

**Published:** 2021-10-20

**Authors:** Haiqin Jiang, Lemuel Tsang, Hongsheng Wang, Changhong Liu

**Affiliations:** ^1^State Key Laboratory of Pharmaceutical Biotechnology, School of Life Sciences, Nanjing University, Nanjing, China; ^2^College of Medicine, University of Tennessee Health Science Center, Memphis, Tennessee, USA; ^3^Institute of Dermatology, Chinese Academy of Medical Sciences and Peking Union Medical College, Nanjing, China

## Abstract

Interferon-induced protein 44-like (IFI44L) gene is a type I interferon-stimulated gene (ISG) that plays a critical role in antiviral activity and constitutes a promising diagnostic marker. However, its precise role and function in tuberculosis have not been unveiled. This study showed that IFI44L acts as an antimicrobial target and positive modulator in human macrophages. Knockdown of IFI44L led to increased *Mycobacterium tuberculosis* intracellular survival. Moreover, IFI44L was significantly upregulated, and it restricted the intracellular survival of *M. tuberculosis* H37Rv strains at 72 h after rifampicin treatment. Individuals with cutaneous tuberculosis (CTB) were found to have significantly higher IFI44L expression after 6 months of rifampicin therapy than after only 1 month. These results demonstrated that IFI44L induced positive regulation and clearance of *M. tuberculosis* from human macrophages. This antimicrobial activity of IFI44L makes it a possible target for therapeutic applications against *M. tuberculosis*.

## 1. Introduction

*Mycobacterium tuberculosis* (*M. tuberculosis*) is one of the most important infectious agents that continuously cause considerable morbidity and mortality globally; approximately 10 million people became newly sick and 1.4 million died in 2019 [1]. Cutaneous tuberculosis is a rare clinical manifestation of *M. tuberculosis* infection, comprising approximately 1%–2% of all tuberculosis cases, and usually presents in the form of scrofuloderma or lupus vulgaris [2–5]. The interaction between macrophages and the Mycobacterium is thought to play a key role in determining the outcome of infection. Mycobacteria are repleted with pathogen-associated molecular patterns, such as bacterial cell-wall peptidoglycan, which has been shown to activate cytokine responses in cultured macrophages [6]. Bacteria is recognized by receptors on the macrophages, and these receptors include toll-like receptors (TLRs), which rapidly signal the presence of the pathogen through the nuclear factor-kB (NF-kB) and mitogen-activated protein kinase (MAPK) pathways to induce the activation of proinflammatory cytokines and limit the intracellular survival of *M. tuberculosis* [7–9]. A range of macrophage genes is involved in initiating an effective immune response, such as the activation of type I IFN gene expression programs, which have been repeatedly shown in human studies to be important in dictating *M. tuberculosis* infection outcomes [10, 11].

Previous studies indicated a promising role of interferon-induced protein 44-like (IFI44L) in antiviral response and antitumour and inflammatory responses on innate immunity and a critical role it plays in the efficient and rapid limitation of viral infections. IFN-I and IFN-II could activate the IFI44L promoter through IFN-stimulated response elements. Cotransfection of the IFI44L promoter with an HIV-1 infection activated IFI44L promoter transcription [12]. *IFI44L* is a type I interferon-stimulated gene (ISG) that belongs to the IFI44 family [13]. IFI44L had modest activity against HCV and inhibited HCV replication by less than 20% when expressed after infection [14]. Overexpression of IFI44 or IFI44L was sufficient to restrict RSV infection early postinfection and control RSV infection [15]. As mentioned earlier, whole-blood transcriptomic RNA signatures discovered in *M. tuberculosis* progressors and healthy controls showed that ISG could largely predict progression from infection to *M. tuberculosis* disease, with promising sensitivity and specificity [16]. *M. tuberculosis* could affect the expression of IFI44L, other ISG genes, and cytokines through regulatory pathways in the macrophages [17]. However, the precise role of IFI44L and its diagnostic prognosis in tuberculosis has not been unveiled.

This study is aimed at determining if IFI44L mediates early innate immunity and prognostic therapy. The results demonstrated that *M. tuberculosis* activates ISG pathways, leading to the upregulation of IFI44L, accompanied by proinflammatory cytokines and chemokine release, which enhanced *M. tuberculosis* clearance in human macrophages. IFI44L could also serve as a quantitative prognostic marker for treatment response in macrophages and individuals with cutaneous tuberculosis, thereby further supporting the role of IFI44L in clearing *M. tuberculosis*. The data suggested that IFI44L is a positive regulator of *M. tuberculosis* clearance and a potential prognostic marker depicting the efficacy of rifampicin treatment.

## 2. Materials and Methods

### 2.1. Ethics Statement

The collection of human blood samples from healthy donors and cutaneous tuberculosis patients at the Institute of Dermatology, Chinese Academy of Medical Sciences, was approved by the ethics committee (2019-KY-013). Written informed consent was obtained from all participants involved in the study.

### 2.2. Blood Sample Collection

Whole blood and tissue samples from registered cutaneous tuberculosis (CTB), skin lesion and parietal tissue specimens (*n* = 10), blood donors (*n* = 10), and healthy donors (*n* = 10) were collected at the Hospital for Skin Diseases, Chinese Academy of Medical Sciences. CTB patients were diagnosed there using previously published criteria [18]. All CTB patients were collected epidemiology database, consisting of patients' age, sex, clinical features, and findings from histopathology, bacteriology, and routine blood and urine tests. Results from PPD screens, HIV antibody analysis, liver and renal function analysis, and chest radiography are included. Patients were HIV-negative and had no other immunocompromising conditions. Rifampicin as a whole-course treatment, the first 2 months of intensive treatment was supplemented with isoniazid, and the next 4 months of maintenance treatment was supplemented with ethambutol. The patients with CTB patients did not only receive rifampicin but also completed the entire course of drug therapy with rifampicin as the mainstay.

### 2.3. PBMC Isolation

Whole blood (5 mL) was collected from each participant into EDTA tubes. Peripheral blood mononuclear cells (PBMCs) were isolated after serum collection using Hyman Lymphocyte Separation Tubes (Dakewe Biotech, China) according to the product's instructions. Protein and RNA were isolated from extracted PBMCs using 100 *μ*L RIPA Lysis Buffer (Invitrogen, USA) and 100 *μ*L TRIzol reagent (Invitrogen, USA) separately according to the manufacturer's instructions.

### 2.4. THP-1 Cell Culture and *M. tuberculosis* H37Rv Strain Infection

Human THP-1 cells (ATCC TIB 202) were obtained from the Institute of Dermatology, Chinese Academy of Medical Sciences. Cells were maintained in RPMI-1640 medium (Gibco, Gaithersburg, MD, USA), which was supplemented with 10% fetal bovine serum and 100 U/mL each of penicillin and streptomycin. Cells were cultured at 37°C in a humidified atmosphere of 5% CO_2_. Prior to infection, the cells were seeded at a density of 1 × 10^5^ cells per mL and differentiated into macrophages using 100 *μ*g/mL phorbol 12-myristate 13-acetate (PMA) for 48 h, followed by a 24-hour incubation in fresh RPMI-1640 medium. Strains of *M. tuberculosis* H37Rv were grown in Middlebrook 7H9 medium with Middlebrook OADC enrichment for 2 weeks (Sigma, St. Louis, MO). *M. tuberculosis* H37Rv infections were carried out for 6 hours at multiplicities of infection (MOIs) of 0, 1, 5, and 10, followed by washing twice to remove extracellular bacteria, and then further incubated in RPMI-1640 with 10% fetal bovine serum for the indicated times [19].

### 2.5. RNA Isolation, Sequence, and Analysis of Host Transcriptomes

For obtaining RNA from infected macrophages, triplicate wells of THP-1 cells were infected with *M. tuberculosis* H37Rv at MOIs of 5 and harvested at three time points, i.e., 6 h, 24 h, and 48 h, as described in the previous section. The washed cells were lysed using the reagents in the QIAamp RNA Mini Kit according to the manufacturer's instructions (Qiagen, Venlo, The Netherlands). RNA quantity and quality were estimated with a Qubit 2.0 Fluorometer and Agilent Technology 2100 Bioanalyzer, respectively. Sequencing libraries were generated using the NEBNext® Ultra™ RNA Library Prep Kit for Illumina® (NEB, USA) following the manufacturer's recommendations, and index codes were added to attribute sequences to each sample. Reference-based maps of host transcriptomes were aligned with HISAT2 [20], while differentially expressed genes (DEGs) were identified using GFOLD (version 1.1.4) [21]. KEGG pathway enrichment analysis was based on Fisher's exact test with 22,810 human protein-coding genes as background by clusterProfiler [22].

### 2.6. Viability of *M. tuberculosis* after Infection with THP-1

Prior to infection, the THP-1 cells were transferred to 24-well plates at 1.5 × 10^5^ cells/well and pretreated with 100 *μ*g/mL phorbol 12-myristate 13-acetate for 48 h to induce differentiation into macrophages. *M. tuberculosis* H37Rv infections were given to the macrophage culture in triplicate wells and incubated for 6 hours at multiplicities of infection (MOIs) of 0, 5, 10, and 20, followed by washing and incubating cells to remove adhered bacteria. Cells were further incubated in RPMI-1640 with 10% fetal bovine serum for the indicated times. At 6, 24, and 48 h, the cells were lysed with 0.05% Tween 20 (Sigma Aldrich) and plated on Löwenstein-Jensen (L-J) medium. Colony-forming units (CFUs) from infected cells were compared with those of the original inoculum of mycobacteria plated directly on L-J medium [19]. The entire experiment was repeated thrice, thereby yielding a total of nine wells that included three wells per species, including mock-infected cells.

### 2.7. Quantitative Real-Time Polymerase Chain Reaction Assay

Total RNA was isolated from THP-1 cells using the TRIzol reagent (Invitrogen, Rockville, MD). The M-MLV First Strand Kit (Taraka, Dalian, China) was used to prepare complementary DNA. Reaction conditions were as follows: 95°C for 10 minutes, followed by 40 cycles of 95°C for 20 seconds, and 56°C for 60 seconds. Gene expression levels for IFI44L were detected by qPCR using an LC480 instrument (primer details are listed in Table 1). Fold differences in expression levels among RNA samples were calculated via the 2-*ΔΔ*ct method after normalization to *β*-actin. Findings were verified by repeating the THP-1 infection experiment, extracting RNA, and performing qPCR for the panel of genes as described previously.

### 2.8. Western Blot Analysis

Macrophages from THP-1 differentiation were lysed with lysis buffer (Cell Signaling Technology, Beverly, MA), and the BCA assay kit (Beyotime, Jiangsu, China) was used to quantitate protein concentrations. Then, 40 *μ*g of protein was separated by 10% sodium dodecyl sulfate polyacrylamide gel electrophoresis and transferred to polyvinylidene difluoride membranes (Millipore, Billerica, MA). Membranes were then blocked and incubated overnight at 4°C with primary antibodies, followed by incubation with HRP-conjugated secondary antibodies for two hours. The ECL reagent (Pierce, Chester, UK) was used to detect the values of band intensities. ImageSaver (ATTO, Tokyo, Japan) was used for densitometry analysis. The primary antibodies used were rabbit anti-IFI44L antibody (Polyclonal, Abgent, San Diego, CA) and mouse anti-GAPDH antibody (Cell Signaling Technology).

### 2.9. Annexin V and PI Staining

THP-1 cells differentiated with PMA were plated into 24-well flat-bottom plates at 5∗10^5^ cells per well, incubated for 6 h with *M. tuberculosis* H37Rv (MOI of 5), and then washed thoroughly. Cells were harvested on successive days and then washed twice with PBS and resuspended in 400 *μ*L of annexin V staining buffer (BD PharMingen, Los Angeles, Calif). One hundred *μ*L of cell suspension was transferred to new tubes, and 5 *μ*L of fluorescein isothiocyanate- (FITC-) conjugated annexin V and 2 *μ*L of propidium iodide were added. Cells were incubated in the dark for 15 min, and then, 400 *μ*L of staining buffer was added. Stained cells were immediately analyzed using a BD FACSVerse flow cytometer and FlowJo10.7 software.

### 2.10. Colony-Forming Unit Assay

THP-1 cells expressing the IFI44L shRNA (or scramble control shRNA) were infected with *M. tuberculosis* H37Rv and incubated for 6 hours at 37°C, after which the extracellular mycobacteria were removed by washing with PBS. The infected cells were cultured for another 12, 24, 48, or 72 hours. After that, quantitative culturing was performed using 10-fold serial dilutions on Middlebrook 7H10 agar plates supplemented with OADC. Plates were cultured for 3 weeks, and colony-forming units (CFUs) were counted.

### 2.11. Detection of Chemokines and Cytokines

For cell supernatants and serums collected at 0 h, 6 h, 12 h, 24 h, 48 h, and 72 h, concentrations of IL18, CCL4, CXCL10, and CXCL11 were tested using commercial ELISA kits according to the manufacturer's protocols (Neobioscience, China).

### 2.12. Statistical Analysis

Statistical significance was determined with GraphPad Prism 5 software. Data analyses were performed using two-tailed *t*-tests. Comparisons between groups were performed using analysis of variance. Data are presented as the mean standard deviation. Significant differences were assigned to *P* < 0.05 (∗), *P* < 0.01 (∗∗), and *P* < 0.001 (∗∗∗).

## 3. Results

### 3.1. IFI44L Was Upregulated in Macrophages during *M. tuberculosis* H37Rv Infection

**Figure 1:**
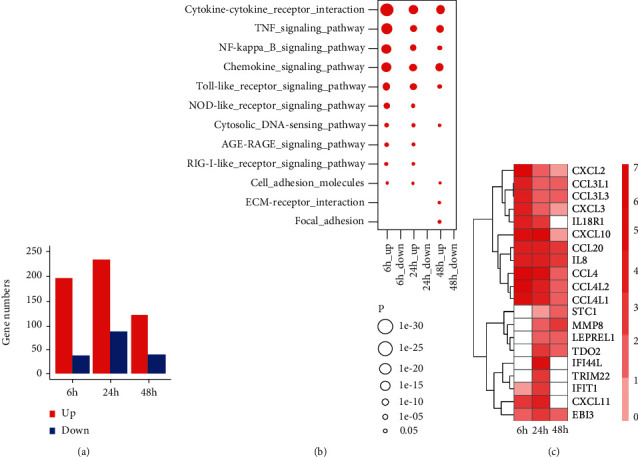
Differential gene analysis for *M. tuberculosis* H37Rv-infected macrophages by RNA-sequence. (a) Number of differentially expressed genes at time points of 6, 24, and 48 h in *M. tuberculosis* H37Rv-infected macrophages. (b) Red circle size means significant enrichment of upregulated genes by KEGG pathway enrichment. Each score represents base 10 to the minus 5 logarithm of enrichment *P* values. (c) Gene expression signature of top 10 upregulated differentially expressed genes from each time point of *M. tuberculosis* H37Rv-infected macrophages. The colour denotes log2 fold change of gene expression. Colour red indicates upregulation and blue means downregulation.

RNA-sequencing (RNA-seq) was performed at the established key innate immune time points of 6, 24, and 48 h [17, 23], and the results showed that more than 200 genes were significantly upregulated at 24 h (Figure 1(a)). Pathway analysis was performed to identify genes/pathways that were induced by *M. tuberculosis* H37Rv, and the major enriched pathways were found to be the cytokine-cytokine receptor interaction, TNF signaling pathway, the NF-kB signaling pathway, and the chemokine signaling pathway (Figure 1(b)). Intensive induction of proinflammatory cytokines (IL8 and IL18), proinflammatory chemokines (CCL4, CXCL10, and CXCL11), and antimicrobial molecules, such as TRIM22, IFIT1, and IFI44L, was observed in macrophages infected with *M. tuberculosis* H37Rv compared with macrophages with no-infection bacteria (Figure 1(c)). In particular, the activation of these immune receptors led to a range of host anti-*M. tuberculosis* immunity, with the strongest at 24 h and a decrease at 48 h. The expression of antituberculous genes could be interpreted as early-host antituberculous immune response and, with further *M. tuberculosis* infection, evasion of host clearance. The differential gene expression by log2 fold change was analyzed for comparing the infected group with the uninfected group (*P*, 0.05). All experiments were repeated for three biological replicates.

**Figure 2:**
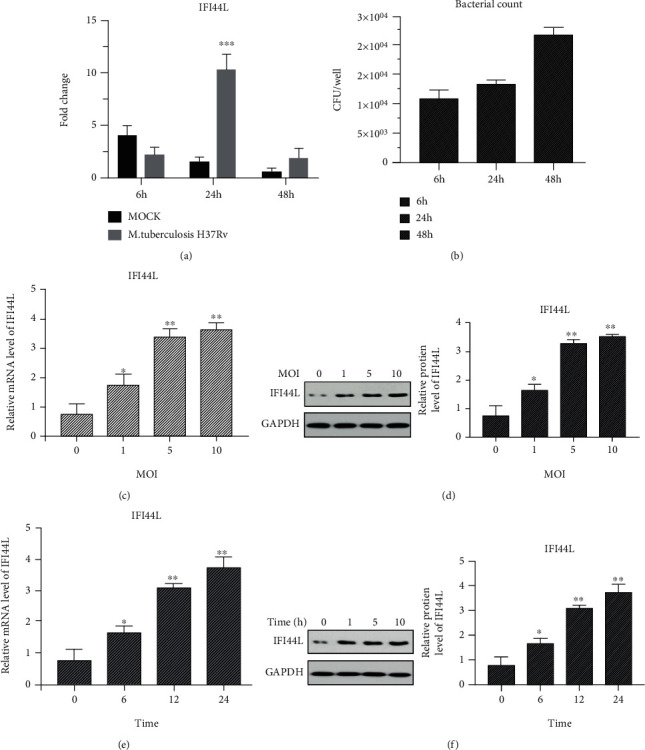
IFI44L induced by *M. tuberculosis* H37Rv-infected macrophages. (a) IFI44L was significantly upregulated at 24 h in *M. tuberculosis* H37Rv-infected macrophage group. (b) Intracellular *M. tuberculosis* H37Rv bacteria were collected in 6, 24, and 48 h for culture in L-J medium. CFU is the number of colony-forming units. (c, d) THP-1 cells infected with *M. tuberculosis* H37Rv at MOIs of 0, 1, 5, or 10 for 24 h. IFI44L expression measured by qPCR and Western blot analysis. (e, f) THP-1 cells infected with *M. tuberculosis* H37Rv at an MOI of 5 for 0, 6, 12, and 24 h and IFI44L level detected by qPCR and Western blot analysis. Values are presented as levels relative to *β*-actin and GAPDH. ^∗^*P* < 0.05, ^∗∗^*P* < 0.01, and ^∗∗∗^*P* < 0.001.

Next, qPCR was used to measure the expression of several important innate immune transcripts in THP-1 to validate the RNA-seq results. The upregulated gene IFI44L belonged to the IFI44 family, which is consistent with themselves being ISGs. We scored CFUs of *M. tuberculosis* H37Rv in THP-1 cells infected with the presence of IFI44L, and the survival of *M. tuberculosis* H37Rv was inhibited within 24 h in THP-1 cells. As the duration of the infection, IFI44L expression was downregulated and the replication ability of the bacteria increased at 48 h (Figures 2(a) and 2(b)). This finding showed that antiviral genes inhibit bacterial proliferation on early innate immunity; as infection progresses, the bacteria escape the host interference. The mRNA sequence datasets were analyzed to evaluate the regulating factor in macrophages during *M. tuberculosis* H37Rv infection. Interestingly, IFI44L, an interferon-induced gene, was upregulated in macrophages 24 h post-*M. tuberculosis* H37Rv infection. Indeed, the RT-PCR results showed that IFI44L expression increased in *M. tuberculosis* H37Rv-infected THP-1 cells in a dose-dependent and time-dependent manner (Figures 2(c) and 2(e)). The Western blot assay reinforced these results, with the level of IFI44L peaking at an *M. tuberculosis* H37Rv dose of MOI and time-dependently (Figures 2(d) and 2(f)). These findings suggested that IFI44L upregulation regulates the innate immune response to infection by *M. tuberculosis*.

### 3.2. Upregulation of IFI44L Promoted Macrophage Differentiation and Facilitated Inflammatory Cytokine Secretion

**Figure 3:**
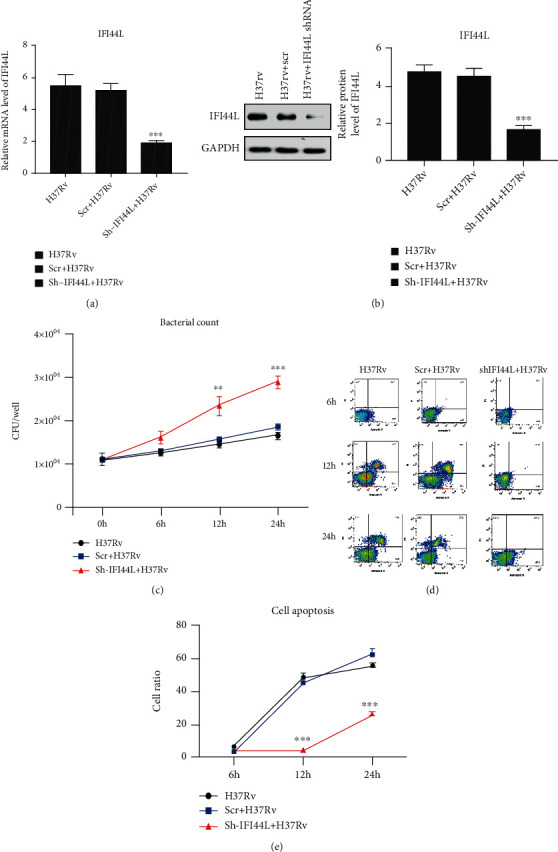
Relative levels of IFI44L and survival of *M. tuberculosis* H37Rv in the presence of shRNA-IFI44L. (a, b) THP-1 cells transfected with IFI44L-specific shRNA (shRNA-IFI44L) and its scramble shRNA (Scr-shRNA) followed by *M. tuberculosis* H37Rv infection. (c) Survival of *M. tuberculosis* H37Rv augmented by CFUs in the presence of shRNA-IFI44L. (d, e) *M. tuberculosis* H37Rv-infected THP-1 cells with shRNA-IFI44L exhibiting significantly decreased cell apoptosis, as shown in flow cytometry. Values are presented as levels relative to *β*-actin. ^∗∗^*P* < 0.01; ^∗∗∗^*P* < 0.001.

**Figure 4:**
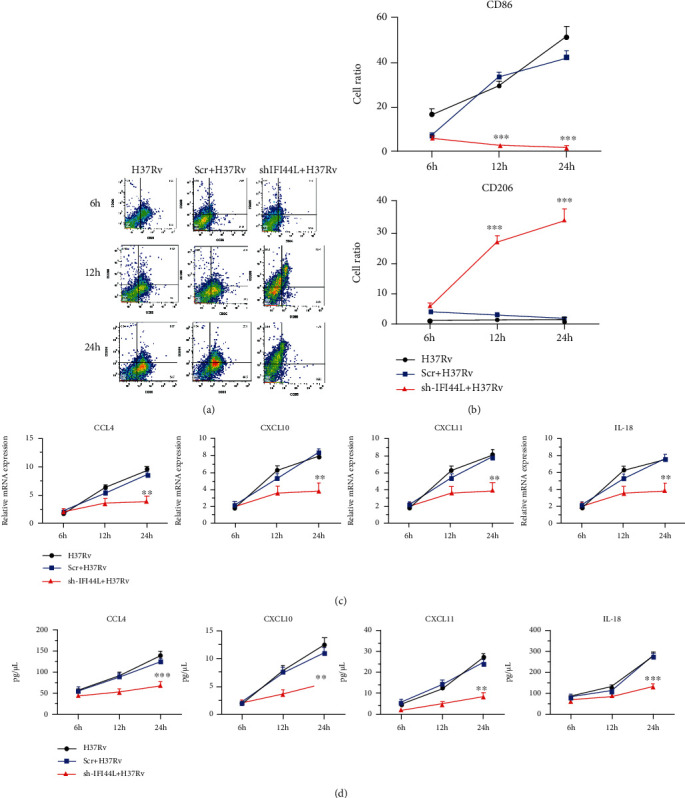
IFI44L promoted macrophage differentiation and inflammatory cytokine secretion. (a, b) CD86 and CD206 expressions analyzed by flow cytometry. (c, d) CCL4, CXCL10, CXCL11, and IL18 expression of *M. tuberculosis* H37Rv-infected cells with shRNA-IFI44L augmented by qPCR and ELISA at 6, 12, and 24 h. Values are presented as levels relative to *β*-actin. ^∗∗^*P* < 0.01; ^∗∗∗^*P* < 0.001.

THP-1 cells were transfected with IFI44L specific shRNA (shRNA-IFI44L) and its scramble shRNA (Scr-shRNA) followed by *M. tuberculosis* H37Rv infection to examine the potential role of IFI44L in infected macrophages (Figures 3(a) and 3(b)). The CFUs in these cells were scored, and the results showed that the survival of *M. tuberculosis* H37Rv was augmented in the presence of shRNA-IFI44L (Figure 3(c)). M1-like macrophage surface marker CD86 and M2-like macrophage surface marker CD206 were observed to analyze the polarization typing of macrophages after bacterial infection. Flow cytometry results showed that *M. tuberculosis* H37Rv-infected THP-1 cells with shRNA-IFI44L exhibited significantly decreased cell apoptosis (Figures 3(d) and 3(e)), while the expression levels of CD86 and CD206 were up- and downregulated, respectively (Figures 4(a) and 4(b)). The result suggested that macrophages were polarized to an M1 proinflammatory phenotype after *M. tuberculosis* H37Rv infection. The qPCR results showed that *M. tuberculosis* H37Rv-infected cells with shRNA-IFI44L had significantly decreased mRNA expression of CCL4, CXCL10, CXCL11, and IL18 compared with the *M. tuberculosis* H37Rv-infected cells without transfection and those with Scr-shRNA at 24 h (*P* < 0.01, Figures 4(c) and 4(d)). By using commercial ELISA kits, the concentrations of proinflammatory cytokines were measured in THP-1 cell culture supernatants that were collected at 6, 12, and 24 h. The results showed that the levels of CCL4, CXCL10, CXCL11, and IL18 were markedly downregulated at 24 h in *M. tuberculosis* H37Rv-infected THP-1 cells with shRNA-IFI44L. Therefore, IFI44L upregulation decreased bacterial survival, promoted the polarization of macrophages and inflammatory cytokine secretion, and may help inhibit infection.

### 3.3. IFI44L Overexpression Restricted the Intracellular Survival of *M. tuberculosis* H37Rv after Rifampicin Treatment

**Figure 5:**
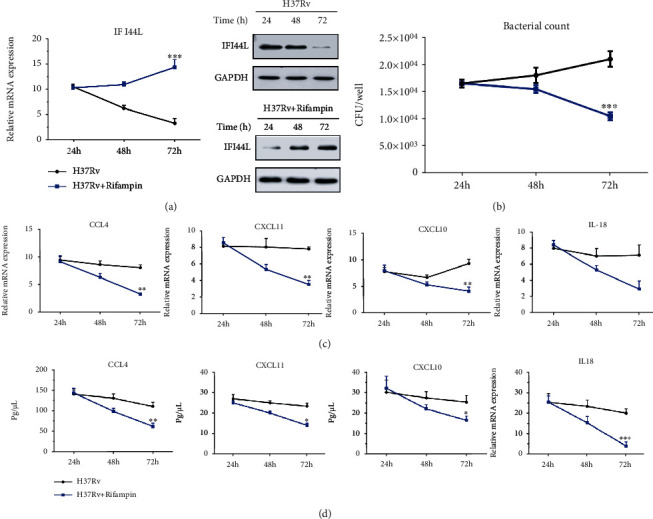
Analysis of inflammatory gene expression after rifampicin treatment in *M. tuberculosis* H37Rv-infected macrophages. (a, b) Increase in IFI44L expression in *M. tuberculosis* H37Rv-infected macrophages after 24 h of rifampicin therapy and significant increase after 72 h of rifampicin therapy in comparison with levels at 24 h. Intracellular survival of *M. tuberculosis* was restricted at 72 h, as shown by CFU counting assays. (c, d) CCL4, CXCL10, CXCL11, and IL18 expressions determined by qPCR and ELISA at 24, 48, and 72 h. Values are presented as levels relative to *β*-actin and GAPDH. ^∗∗^*P* < 0.01; ^∗∗∗^*P* < 0.001.

THP-1 cells were infected with *M. tuberculosis* H37Rv followed by treatment using rifampicin at 24 h. Then, IFI44L expression and bacterial count were further observed at 48 and 72 h. IFI44L expression increased in *M. tuberculosis* H37Rv-infected macrophages after rifampicin therapy and increased significantly after 72 h of rifampicin therapy in comparison with the levels at 24 h. The intracellular survival of *M. tuberculosis* H37Rv was restricted at 72 h as shown in CFU counting assays (Figures 5(a) and 5(b), *P* < 0.01). By contrast, the expression levels of CCL4, CXCL10, CXCL11, and IL18 decreased significantly during the same timeframe (Figures 5(c) and 5(d), *P* < 0.01). In other words, the expression of these genes decreased significantly after 72 h of treatment compared with that after 24 h of rifampicin therapy. These data demonstrated that the significant decrease in CCL4, CXCL10, CXCL11, and IL18 expression and increase in IFI44L expression observed in *M. tuberculosis* H37Rv-infected macrophages after rifampicin treatment at 72 h may be associated with a decreased risk of *M. tuberculosis* infection.

### 3.4. Increased IFI44L Expression in Individuals of Cutaneous Tuberculosis (CTB) after Rifampicin as Main Drug Therapy

**Figure 6:**
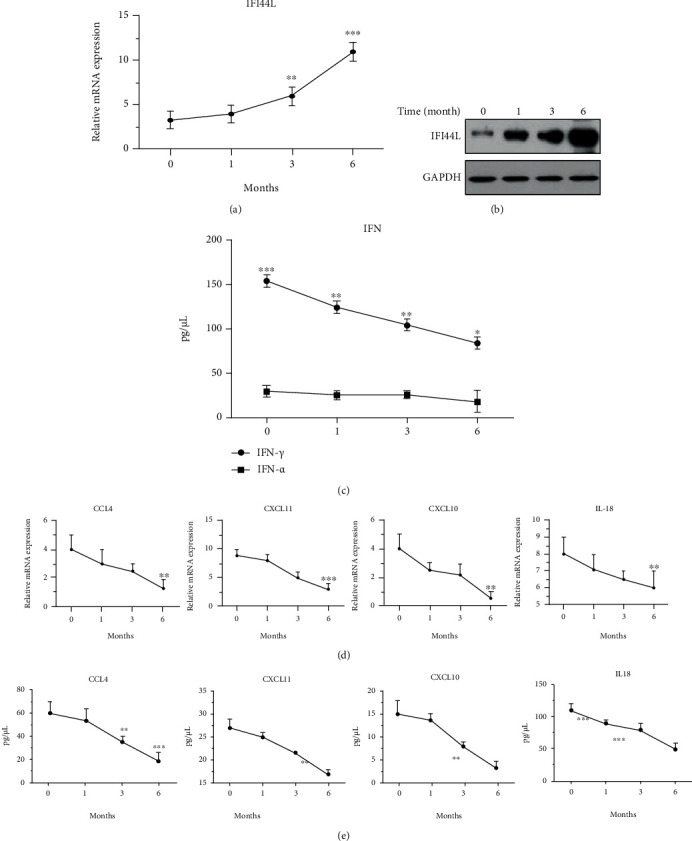
Relative mRNA levels of IFI44L, CCL4, CXCL10, CXCL11, and IL18 PBMC from individuals with CTB after rifampicin as main drug treatment. (a, b) IFI44L expression determined by qPCR and Western blot for 1, 3, and 6 months. (c) IFN-*α* and IFN-*γ* expression determined by ELISA. (e, f) CCL4, CXCL10, CXCL11, and IL18 expression determined by qPCR and ELISA. Values are presented as levels relative to *β*-actin and GAPDH. ^∗∗^*P* < 0.01; ^∗∗∗^*P* < 0.001.

The fold change in gene expression of the above genes after rifampicin as the main drug treatment was examined for individuals with CTB to study the relationship between the long-term effects of antituberculosis treatment and *IFI44L* expression. qPCR and ELISA were used to analyze the relative expression levels of IFI44L, CCL4, CXCL10, CXCL11, and IL18 mRNAs and proteins in PBMC samples. The results after 0 days and 1, 3, and 6 months of antituberculosis therapy were compared. IFI44L expression significantly increased in CTB individuals after 3 and 6 months of antituberculosis therapy compared with the levels before antituberculosis therapy (Figures 6(a) and 6(b)). The key molecules IFN-*α* and IFN-*γ* stimulated by IFN-I and IFN-II were analyzed. IFN-*γ* was significantly upregulated in the course of antituberculosis treatment (Figure 6(c)). By contrast, CCL4, CXCL10, CXCL11, and IL18 expression significantly decreased among all individuals after 1 month of antituberculosis therapy, and the decrease was significantly higher among the same individuals after 6 months of antituberculosis therapy (Figures 6(c) and 6(d)). The significant changes in the expression of IFI44L, IFN-*γ*, and cytokine genes among individuals with CTB were associated with innate host immunity against *M. tuberculosis*.

## 4. Discussion

This study showed that IFI44L acted as an antimicrobial target and a positive modulator in human macrophages. Knockdown of IFI44L led to the increased intracellular survival of *M. tuberculosis* H37Rv and the decrease in inflammatory cytokine secretion. Moreover, after *M. tuberculosis* H37Rv strains infected with THP-1 were treated with rifampicin from 24 h to 72 h, IFI44L was significantly upregulated and it restricted the intracellular survival of these strains. In individuals of CTB, IFI44L expression was found to significantly increase after several months of rifampicin as the main drug therapy. The results demonstrated that IFI44L induced positive regulation and clearance of *M. tuberculosis* from human macrophages. This antimicrobial activity of IFI44L makes it a possible target for therapeutic strategies against *M. tuberculosis*.

The function and pathway enrichment analyses of *M. tuberculosis* H37Rv-infected macrophage by RNA-seq demonstrated that gene enrichment was closely associated with viral infection genes, including TRIM22, IFIT1, IFI44L, and IFI44, consistent with *M. tuberculosis* being an intracellular pathogen. These antiviral genes involved in the innate immune response as part of the host defence response to clear viral infections were specifically upregulated, including IFI44L and TRIM22 in *M. tuberculosis*-infected macrophages [17, 19]. The expression of IFI44L family members was activated by bacteria and viruses in human THP-1-derived macrophages [24]. IFI44L is an interferon-induced protein localised to the cytoplasm, and it is upregulated by IFN regulatory factor by binding to one of the IFN-stimulated response elements [12]. In a tumour disease, IFI44L activated the met/Src signaling pathway and had effects on cancer stemness, metastasis, and drug resistance [25]. However, whether IFI44L regulates macrophage behaviour during *M. tuberculosis* infection needs further study. In the current study, IFI44L expression was significantly upregulated, and it led to antituberculosis responses in THP-1 cells infected with *M. tuberculosis* H37Rv.

The data showed that IFI44L knockdown reduced cytokine and chemokine secretion, leading to an inhibition of the inflammatory response, whereas the increased IFI44L expression promoted proinflammatory response in THP-1 cell lines. IFI44L upregulation enhanced CD86, CCL4, CXCL10, CXCL11, and IL18 expression and led to the inhibition of *M. tuberculosis* activity in THP-1 immune cells. A recent study indicated a significant upregulation of IFI44L involved in *M. tuberculosis* infection of THP-1 cells [17]. In addition, IFI44L was proposed as a positive suppressor modulating the anti-inflammatory response. CCL4, CXCL10, CXCL11, and IL18 expression was significantly decreased after 24 h of rifampicin treatment and after 1 month of antituberculosis therapy in CTB individuals. CXCL10 has been used to distinguish between tuberculosis and latent tuberculosis [26], and a significant decrease in CCL4 expression was observed in individuals with latent tuberculosis after antituberculosis treatment associated with a decreased risk of developing active tuberculosis [27]. This result suggested that 6 months of rifampicin as the main drug treatment negatively regulates CCL4, CXCL10, CXCL11, and IL18. These gene expression patterns observed in individuals after antituberculosis treatment may be associated with a decreased risk of developing active tuberculosis and could be useful as biomarkers of treatment efficacy.

Further study identified that IFI44L was upregulated at 72 h in THP-1 cells after rifampicin therapy. In addition, IFI44L expression increased significantly after 6 months compared with 1 month of rifampicin as the main drug treatment among individuals with CTB. IFN-*γ* was significantly upregulated in CTB-positive individuals in the course of antituberculosis therapy. Therefore, IFI44L expression is stimulated by IFN-*γ* of IFN-II in CTB-positive individuals. Some prevalent biosignatures in the blood of patients with tuberculosis are the elevated expression of transcripts involved in type II IFN signaling [28]. Carneiro et al. found that high IFN-*γ* upregulates the expression of CCL7, IL8, IFI44L, and IL1*β* associated with cutaneous leishmaniasis [29]. It has been described that various stimuli, such as infection with viruses or viral and bacterial molecular patterns, have been shown to directly induce transcription of the IFIT1/ISG56 family [30]. Other authors have reported that the IFI44L expression in IGRA+ and IGRA− patients was significantly upregulated after 6 months of isoniazid treatment [31]. Therefore, mycobacterial antigens could be continuously released during antituberculosis treatment and induce the increasing IFI44L expression. So, our observation after 24 h of rifampicin treatment and 3 months of rifampicin as the main drug treatment indicated that IFI44L expressions might be relevant as biomarkers of the effectiveness of antituberculosis therapy. These results also support the suitability of prolonged rifampicin as the main drug administration. Moreover, IFI44L expression in individuals with CTB potentially represents an approach to monitor patient response to rifampicin as the main drug treatment.

In conclusion, IFI44L was significantly increased in THP-1 cells during the standard innate immune response of cells a few days following drug treatment and in individuals with CTB after rifampicin as the main drug therapy. IFI44L expression notably increased after 3 months of antituberculosis therapy, whereas CCL4, CXCL10, CXCL11, and IL18 expression were decreased. These results indicated that FI44L is a prospective biomarker for the effectiveness of antituberculosis therapy.

## Figures and Tables

**Table 1:** Primers for qPCR.

Genes	Direction	Sequence (5′‐3′)
IFI44L	Forward	TGCACTGAGGCAGATGCTGCG
	Reverse	TCATTGCGGCACACCAGTACAG
IFI44L shRNA	Forward	CCGGAGGATAACCTAGACGACATAACTCGAGTTATGTCGTCTAGGTTATCCTTTTTTG
	Reverse	AATTCAAAAAAGGATAACCTAGACGACATAACTCGAGTTATGTCGTCTAGGTTATCCT
Scramble (Scr)	Forward	CCGGCCTAAGGTTAAGTCGCCCTCGCTCGAGCGAGGGCGACTTAACCTTAGGTTTTTG
	Reverse	AATTCAAAAACCTAAGGTTAAGTCGCCCTCGCTCGAGCGAGGGCGACTTAACCTTAGG
CCL4	Forward	GCTTCCTCGCAACTTTGTGG
	Reverse	TCACTGGGATCAGCACAGAC
CXCL10	Forward	CCTGCAAGCCAATTTTGTCCA
	Reverse	TGCATCGATTTTGCTCCCCT
CXCL11	Forward	GAGTGTGAAGGGCATGGCTA
	Reverse	ATGCAAAGACAGCGTCCTCT
IL18	Forward	TGCCAACTCTGGCTGCTAAA
	Reverse	TTGTTGCGAGAGGAAGCGAT
*β*-Actin	Forward	ACAGAGCCTCGCCTTTGCC
	Reverse	ACAGAGCCTCGCCTTTGCC

## Data Availability

The (figures and table) data used to support the findings of this study are included within the article.
